# Barriers and facilitators to using patient-reported experience measures for diabetes care: a qualitative study in Thailand

**DOI:** 10.3389/frhs.2026.1799682

**Published:** 2026-04-10

**Authors:** Soe Sandi Tint, Wichuda Jiraporncharoen, Nida Buawangpong, Myo Zin Oo, Kittipan Rerkasem, Kanokwan Kulprachakarn, Hataichanok Chuljerm, Timothy E. O'Brien, Rohini Mathur, Petch Rawdaree, Chaisiri Angkurawaranon

**Affiliations:** 1 Global Health and Chronic Conditions Research Center, Chiang Mai University, Chiang Mai, Thailand; 2 Department of Family Medicine, Faculty of Medicine, Chiang Mai University, Chiang Mai, Thailand; 3 School of Health Sciences Research, Research Institute for Health Sciences, Chiang Mai University, Chiang Mai, Thailand; 4 Research Institute for Health Sciences, Chiang Mai University, Chiang Mai, Thailand; 5 Clinical Surgical Research Center, Department of Surgery, Faculty of Medicine, Chiang Mai University, Chiang Mai, Thailand; 6 Environmental-Occupational Health Sciences and Non-Communicable Diseases Research Center, Research Institute for Health Sciences, Chiang Mai University, Chiang Mai, Thailand; 7 Department of Mathematics and Statistics, Loyola University Chicago, Chicago, IL, United States; 8 Wolfson Institute for Population Health, University of London, London, United Kingdom; 9 Faculty of Medicine Vajira Hospital, Navamindradhiraj University, Bangkok, Thailand; 10 Diabetes Association of Thailand, Bangkok, Thailand

**Keywords:** barriers and facilitators, patient-reported experience measures (PREMs), primary care, qualitative research, type 2 diabetes mellitus

## Abstract

**Background:**

Patient-reported experience measures (PREMs) provide valuable insights into care quality from the patient's perspective and are particularly relevant for chronic conditions such as type 2 diabetes mellitus (T2DM). However, evidence on their implementation in primary care settings of low- and middle-income countries (LMIC) remains limited.

**Objective:**

This study explored barriers and facilitators to implementing a diabetes-specific PREM in routine outpatient care in a district-level hospital setting in northern Thailand, to inform strategies for its feasible and sustainable use in similar resource-constrained settings.

**Methods:**

A qualitative study was conducted using in-depth interviews and a group interview with patients aged 20 years and older living with T2DM, healthcare providers, and the hospital executive team. A total of 25 participants were purposively selected from a hospital-based non-communicable disease (NCD) clinic. Data were collected using a semi-structured interview guide informed by the Consolidated Framework for Implementation Research (CFIR) and analysed using inductive thematic analysis. Patient demographic data were analysed descriptively.

**Results:**

Among the 19 patients included, the mean age was 59.7 years (SD = 11.9), with a range of educational and occupational backgrounds. Thematic analysis revealed three overarching themes influencing PREM implementation: patient-related factors, instrument characteristics, and contextual and implementation factors. Key facilitators included patients' perceived value of the PREM, trust in healthcare providers, and supportive organizational structures that enable workflow integration and staff allocation. Major barriers were limited literacy, functional constraints among older adults, and challenges related to instrument wording, format preferences, and insufficient resources for staff assistance.

**Conclusion:**

Addressing these factors through tailored questionnaires, flexible administration, and institutional commitment can support the routine collection of patient-reported experiences, strengthen patient-centred care, and inform implementation strategies in similar primary care settings across Southeast Asia.

## 1 Introduction

Patient-centred care has increasingly become a keystone of high-quality healthcare delivery (1). The Agency for Healthcare Research and Quality (AHRQ) identifies patient-centredness as a key dimension of quality, emphasizing care that respects and responds to individual preferences, needs, and values (1). In addition, the World Health Organization (WHO) emphasizes integrated, people-centred health services as essential to achieving equitable access to safe, effective, and high-quality care aligned with individuals' needs (2). This approach promotes coordination of care that respects patient preferences, supports meaningful participation in health decisions, optimizes resource use, and strengthens health system resilience to respond to public health challenges effectively (2). By enhancing communication and partnership between patients and providers, patient-centred care not only improves satisfaction but also leads to better health outcomes, especially in chronic disease management, such as diabetes mellitus (3). To ensure that patient-centred care is delivered effectively, healthcare systems increasingly rely on systematic questionnaires that capture patients’ experiences of care, such as patient-reported experience measures (PREMs), which have been widely studied in primary care settings for their role in quality assessment and improvement (4). These questionnaires help translate the principles of patient-centred care into measurable aspects of healthcare delivery, providing actionable insights to improve quality and inform policy (5).

One such tool is the Patient-Reported Experience Measure (PREM), which is specifically designed to reflect patients' perspectives on aspects such as communication, respect, shared decision-making, and coordination of care (6). Although PREM is closely related to patient satisfaction, it differs in that it asks patients to report specific aspects of their care experience rather than evaluate how satisfied they were. In other words, PREMs provide more actionable insights into care processes, whereas satisfaction surveys tend to reflect subjective evaluations and expectations (7).

Several high-income countries have integrated both general and disease-specific PREMs into routine healthcare quality assessment and policy-making (8, 9). General PREMs provide valuable insights into patients' overall care experiences (10), while disease-specific PREMs offer more targeted information, particularly relevant for chronic conditions such as diabetes mellitus (11). However, despite this, the use of PREMs remains limited in low- and middle-income countries (LMICs) (6), often due to resource constraints, differences in health system organization, and cultural factors that require careful adaptation of these tools (12). A recent systematic review identified only 12 studies describing 14 tools developed in LMICs, and among them, only seven assessed patient experiences, while the remainder focused on patient satisfaction (13). Disease-specific PREMs were even less common; for example, one study from South Africa reported the development of a PREM for substance abuse treatment services (14). Although some LMICs have begun exploring both general and disease-specific PREMs, no diabetes-specific PREMs have been developed or used in primary care settings in Southeast Asia, as highlighted by recent literature (15, 16). To address this gap, our team recently developed a diabetes-specific PREM tailored for use in primary care settings in the Thai context (17). While this development marks an important step forward, understanding how to successfully implement such tools in real-world settings remains a critical challenge.

Previous studies from high-income countries, such as the United Kingdom and Sweden, have identified several challenges in implementing PREMs, including staff workload, difficulties in interpreting data, and integration into existing clinical workflows (18). However, these findings may not be directly applicable to LMICs, where health systems often face different contextual realities, such as constrained resources, lower levels of digital infrastructure, and differing patient–provider relationships. These differences highlight the need for context-specific evidence to guide the effective implementation of PREMs in LMIC settings. Therefore, this study aims to explore the barriers and facilitators to implementing our diabetes-specific PREM in routine primary care in a Thai context, providing essential insights that may inform future adoption and scale-up across similar Southeast Asian settings.

## 2 Methods

### 2.1 Study design

This study employed a qualitative case study design to explore the implementation of a diabetes-specific PREM in routine primary care (19). The study was informed by an interpretivist theoretical position (19), recognizing that barriers and facilitators to PREM implementation are understood through the perspectives of patients, healthcare providers, and hospital executives, and are shaped by their roles and organizational context.

Data were collected through semi-structured in-depth interviews and one group interview with multi-level participants involved in diabetes care, including adults with T2DM, healthcare providers directly engaged in diabetes management, and members of the hospital executive team. Including diverse participant groups is considered essential in implementation research to capture contextual factors influencing the integration of new interventions into routine practice (20–22).

The study was reported in accordance with the Standards for Reporting Qualitative Research (SRQR) guidelines, which outline 21 items essential for transparent and rigorous reporting of qualitative research (23).

### 2.2 Study setting

This study was conducted at a district-level hospital-based non-communicable disease (NCD) clinic located in Saraphi District, Chiang Mai, Thailand. The clinic provides outpatient diabetes services twice weekly. Each clinic session lasts approximately 4–5 h and serves around 60 patients per session.

The multidisciplinary care team typically includes one physician, 2–3 nurses, a pharmacist, and supporting administrative staff. For this study, healthcare providers directly involved in diabetes management and clinical decision-making were invited to participate, including the physician, nurse head, nurse assistant, and case manager. The clinic manages approximately 1,800 adults living with type 2 diabetes mellitus (T2DM). Most patients are Thai nationals aged between 30 and 70 years. Patients with more complex conditions, such as type 1 diabetes or severe complications, are referred to higher-level hospitals for specialized care.

Patients with T2DM generally attend scheduled follow-up appointments every three months for routine monitoring of glycemic control, medication adjustment, and lifestyle counseling. Visits are primarily scheduled rather than *ad hoc*, although occasional unscheduled visits may occur for acute concerns. During a typical visit, patients undergo vital sign measurement and laboratory review before consulting with a physician or nurse for ongoing management.

### 2.3 Population and sampling

This study focused on three key groups involved in diabetes care at a district-level, hospital-based NCD clinic: adult patients with type 2 diabetes mellitus receiving routine outpatient services, healthcare providers directly involved in diabetes management, and members of the hospital executive team responsible for overseeing clinical operations. A purposive sampling strategy was used to recruit participants with relevant experiences and roles in diabetes care. This approach allowed the inclusion of individuals able to provide diverse perspectives on the implementation and potential use of PREMs within routine clinical practice. Efforts were made to include patients with varying educational and demographic backgrounds to capture a range of experiences.

### 2.4 Inclusion and exclusion criteria

Patients were eligible to participate if they were 20 years or older, had been receiving outpatient care at the study site for at least 12 months, were able to communicate their experiences, and provided written informed consent. Patients were excluded if they were enrolled in another clinical trial, had a severe illness that limited their ability to participate, or were unable to communicate verbally. The PREM was introduced as a pilot initiative and was not distributed universally to all clinic patients during the study period. Instead, purposive sampling was used to recruit eligible patients who could provide diverse perspectives on its implementation. Efforts were made to include patients with varying educational and demographic backgrounds to capture a range of experiences. Consenting participants were invited to complete the pilot PREM during their routine clinic visit.

Healthcare providers and hospital executive members were eligible if they had been involved in delivering or supervising diabetes care at the study site within the past 12 months and provided written informed consent. Individuals in this group were excluded if they were currently participating in another clinical trial.

### 2.5 Sample size

A total of 25 participants took part: 19 patients, three healthcare providers, and three hospital management members. The sample size was guided by the aim to capture a comprehensive range of stakeholder perspectives. Previous research suggests that 12–20 interviews are typically enough to reach thematic saturation in relatively homogeneous populations (24). This sample was therefore considered adequate to address the study's objectives.

### 2.6 Study procedures

#### 2.6.1 PREM pilot administration

During the study period, the PREM was introduced as a pilot initiative within the NCD clinic to assess its feasibility and explore potential barriers and facilitators to routine implementation. The instrument, which had previously undergone psychometric testing (17), was not re-validated in this study. Instead, the focus was on examining its acceptability and practical integration into routine diabetes care.

The PREM was administered in paper format to eligible adult patients with type 2 diabetes attending scheduled follow-up appointments. Patients who provided consent completed the questionnaire during waiting periods before consultation, which required approximately 10–15 min. Questionnaires were returned to the research team before the clinical encounter. The PREM was administered only to patients meeting predefined inclusion criteria and agreeing to participate; it was not distributed to all clinic attendees. Responses were not reviewed by clinicians during the same visit and were not used to inform individual care decisions, as the instrument was implemented solely for exploratory research purposes.

#### 2.6.2 Qualitative data collection

Data were collected through semi-structured in-depth interviews with patients and healthcare providers, and one group interview with hospital executive members. These discussions explored experiences with diabetes care and perceptions of the PREM pilot initiative. All interviews and the group interview were audio-recorded with participants' consent and transcribed verbatim.

### 2.7 Interview procedures

#### 2.7.1 In-depth interviews

In-depth interviews were conducted with two participant groups (1): patients diagnosed with type 2 diabetes mellitus, and (2) healthcare providers involved in diabetes care at the targeted hospital. Interviews were conducted in Thai in a private consultation room within the outpatient clinic and lasted approximately 30 min.

The semi-structured interview guide was developed based on existing literature on barriers and facilitators to implementing PREMs in diabetes care and was informed by the Consolidated Framework for Implementation Research (CFIR) (25) to ensure coverage of key domains, including (1) intervention characteristics (2), outer setting, inner setting (3), characteristics of individuals, and (4) implementation process. The guide was reviewed by qualitative research experts and the data collection team, then translated and culturally adapted into Thai through an iterative process to ensure clarity and relevance for participants.

Separate interview guides were developed for patients, healthcare providers, and hospital executives to reflect their distinct roles and perspectives within the implementation context. Although the guides shared common implementation domains, questions were tailored to each group to ensure relevance. Patient interviews focused on clarity, usability, perceived usefulness, and potential impact on care experiences, as patients are the end-users of the PREM. Healthcare provider interviews emphasized workflow integration, feasibility, resource availability, and clinical applicability. Executive interviews explored organizational readiness, leadership engagement, policy alignment, and strategic considerations related to adoption and sustainability.

Interviews were conducted by trained bilingual research assistants**.** Research assistants conducted patient and provider interviews to reduce potential power imbalances between participants and senior clinical researchers, thereby encouraging more open discussion of experiences and perceptions. Before data collection, research assistants received training in qualitative interviewing techniques, study protocol procedures, ethical considerations, and the use of the interview guide. Although the interview guides were not formally pilot tested with external participants, they were reviewed and rehearsed within the research team before data collection. Mock interviews were conducted to assess clarity and flow, and minor refinements were made before finalization. Senior members of the research team included clinicians and qualitative researchers with experience in health systems and diabetes care, who provided oversight and contributed to data interpretation.

#### 2.7.2 Group interview

The group interview was conducted with members of the hospital executive team responsible for overseeing the diabetes clinic. The interview was held in a private meeting room within the targeted hospital and lasted approximately 60–90 min. The session aimed to explore administrative and institutional perspectives on the implementation and use of PREMs in routine care. To ensure professional rapport and credibility with high-ranking participants, the session was moderated by two senior members of the research team with experience in qualitative research and health system leadership. The same CFIR-informed guide was adapted to reflect managerial roles, facilitating open discussion on organizational and institutional factors relevant to PREM implementation. The original English and Thai versions of the interview guides were provided as Supplementary Material S1.

### 2.8 Invitation and consent procedures

Eligible patients were approached upon arrival for their scheduled outpatient visit by a designated clinic nurse or coordinator prior to their consultation. Interested individuals were referred to a trained research assistant, who provided a written information sheet explaining the study's purpose, procedures, risks, and benefits. Written informed consent was obtained before participation.Healthcare professionals, including the hospital executive team, were invited by a senior staff member or project coordinator in–person. The invitation included an information sheet describing study objectives, expected time commitment, and data collection procedures. Written informed consent was obtained before participation in the interviews.

All participants were assured that data would be treated confidentially, participation was voluntary, and they could withdraw at any time without consequences for their care or professional standing.

### 2.9 Data management and confidentiality

Audio recordings were transcribed verbatim in Thai by the research assistants who conducted the interviews. Transcripts were subsequently translated into English by a bilingual member of the research team. All transcripts were anonymized by removing identifying information and assigning unique participant codes. Audio files and transcripts were stored on password-protected institutional computers accessible only to authorized members of the research team. Data were managed in accordance with institutional data protection policies and will be securely retained for the period required by the approving ethics committee before permanent deletion.

### 2.10 Data analysis

The data were analysed using inductive thematic analysis (26, 27) to identify patterns and themes related to participants' perceptions and the potential use of the PREM in routine practice. Analysis followed a systematic six-step approach: familiarization with the data, generation of initial codes, searching for themes, reviewing themes, defining and naming themes, and reporting with illustrative quotations. Coding was manually using Microsoft Excel to organize and manage data. To enhance rigor, three researchers (SST, WJ and NB) independently coded a subset of transcripts, with discrepancies resolved through discussion. Themes and sub-themes were further refined through team discussions involving clinicians, qualitative experts, and patient representatives to ensure credibility and relevance.

Trustworthiness was strengthened through data triangulation across three stakeholder groups, peer debriefing among research team members, independent coding, maintenance of an audit trail, and inclusion of verbatim quotations to support interpretations.

Peer debriefing was conducted among members of the research team to discuss coding decisions and theme development, helping to minimize individual bias. Finally, including participants' quotations supported the interpretations, enhancing the confirmability of the results. Participants' characteristics were summarized using frequencies and percentages.

## 3 Results

### 3.1 Socio-demographic data of the patient group

Table 1 describes the characteristics of the patient group. Among 19 patients, the mean age of participants was 59.7 years (SD = 11.9), with ages ranging from 33 to 77 years. Slightly more than half of the participants were male (52.6%), while 47.4% were female. In terms of education, the majority (57.8%) had completed primary school, followed by 31.6% who had attained high school education. Only a small proportion had completed secondary school (5.3%) or held a bachelor's degree (5.3%). Regarding occupation, the majority of participants were classified as employed (52.6%), followed by unemployed (42.1%), while a small proportion were categorized as other (5.3%).

**Table 1:** Socio-demographic characteristics of patient group.

Characteristics of patients		Number	Percentage (%)
Patients (*n* = 19)	**Age (years)**		
	Mean ± SD	59.7 ± 11.9	
	(min – max)	33–77	
	**Gender**		
	Female	9	47.4%
	Male	10	52.6%
	**Education Level**		
	Primary school	11	57.8%
	Secondary school	1	5.3%
	High school	6	31.6%
	Bachelor	1	5.3%
	**Occupation** ^a^		
	Unemployed	8	42.1%
	Employed	10	52.6%
	Other	1	5.3%
Healthcare Providers (*n* = 3)	**Age (years)**		
	Mean ± SD	40 ± 10	
	(min – max)	30–55	
	**Gender**		
	Female	3	100%
	**Occupation**		
	Case manager	1	33.3%
	NCD clinic Nurse	1	33.3%
	NCD clinic Aide	1	33.3%
Executive Team (*n* = 3)	**Age (years)**		
	Mean ± SD	50 ± 5	
	(min – max)	43–52	
	**Gender**		
	Female	2	66.6%
	Male	1	33.4%
	**Occupation**		
	NCD clinic doctor	1	33.3%
	OPD doctor	1	33.3%
	Head nurse	1	33.3%

^a^ Occupational categories were aggregated to broader groups to reduce the risk of participant re-identification. “Unemployed” includes unemployed individuals, housewives, and retired government officials. “Employed” includes private company employees, general employees, individuals running their own business, and sellers. “Other” includes agricultural workers (farmers).

### 3.2 Professional background of healthcare providers and hospital executive team

The study included three healthcare providers: a case manager, a nurse, and a nurse aide from the NCD clinic. The hospital management team consisted of three members: one doctor from the NCD clinic, one doctor from the outpatient department, and the head nurse responsible for hospital-wide nursing services. All participants from these groups were actively involved in both clinical service delivery and administrative decision-making.

### 3.3 Overview

The analysis of interviews with 19 patients, three healthcare providers (HCPs), and three hospital executives identified three overarching themes that influenced perceptions and potential use of the PREM in routine practice: (1) Patient-Related Factors, (2) Instrument Characteristics, and (3) Contextual and Implementation Factors. Key facilitators included patients' perceived value of the PREM, trust in healthcare providers, and supportive organizational structures enabling workflow integration and staff allocation. Major barriers were limited literacy, functional constraints among older adults, and challenges related to instrument design, format preferences, and insufficient staff support. Within each theme, several sub-themes emerged, highlighting both facilitators and barriers to effective implementation. The detailed themes and sub-themes are summarized in Table 2.

**Table 2:** Main themes, Sub-themes and quotes about perceptions and potential use of PREM.

	Main theme	Sub-theme and quotes
1	Patient-Related Factors	**Health Literacy and Comprehension**
		*“I understood most parts because I already know a bit about diabetes” (Patient 5)*
		*“If patients are less educated, they may not understand what goals are” (Executive 2).*
		**Functional Ability and Independence**
		“*I can’t see it clearly; my eyesight is not good. I can’t do it myself” (Patient 3).*
		“*We need to plan for support staff or alternative formats for those who can’t complete it themselves” (Executive 1).*
		**Perceived Value and Usefulness**
		*“It helps me understand more about diabetes, such as how to use my medication properly” (Patient 1*),
		**Trust in the Healthcare Environment**
		*“I’m not worried; it’s general feedback and doesn’t identify me” (Patient 1).*
		*“I trust the professionalism of the healthcare staff” (Patient 6)*
2	Instrument Characteristics	**Design and Usability**
		*“I’m not sure which channel to choose, but having someone at the hospital to help makes it easier” (Patient 19)*.
		**Content Relevance and Applicability**
		*“I never talked to my doctor about setting goals, so that question didn’t apply to me” (Patient 4).*
		“*They don’t understand what it means. They only know their illness, how to treat it, how to take care of themselves, and how to behave” (Executive 2)*
3	Contextual and Implementation Factors	**Feasibility of Routine Implementation**
		*“It’s feasible if we integrate it into waiting times, but not during busy clinic hours” (HCP 3).*
		“*Two visits per year would be fine, because patients come in regularly anyway” (Executive 2)*
		**Policy and Organizational Support**
		*“We need policies that support using this tool for patient-centred quality improvement.” (Executive 3)*

The following sections present the results by overarching theme, with each sub-theme illustrated by selected quotations from participants to provide context and depth to the findings.

### 3.4 Theme 1: patient-related factors

Participants identified key factors that include (1) health literacy and comprehension of the questions, (2) physical and functional ability to complete the form independently, (3) perceived value of the questionnaire, and (4) level of trust in the healthcare environment. These elements collectively determine a patient's engagement with the instrument.

#### 3.4.1 Sub-theme 1.1: health literacy and comprehension

Health literacy and comprehension were key factors influencing patient engagement with the PREM. Most patients perceived the language as clear and straightforward, often linking this to their familiarity with diabetes or prior exposure to health-related materials. For example, one participant explained, “I understood most parts because I already know a bit about diabetes” (Patient 5). Another described the questionnaire as “easy to understand,” attributing this to receiving health education from a family member working in healthcare (Patient 7). Similarly, a participant reported a “medium understanding” because the format resembled questionnaires previously encountered (Patient 1).

However, some patients experienced comprehension difficulties, particularly when encountering medical terminology or lengthy question wording. One patient noted, “I am not used to the medical vocabulary” (Patient 4), while another explained that a question was “a bit long” and required multiple readings to ensure understanding (Patient 6).

Healthcare providers similarly observed that patients with limited literacy often required clarification or support to complete the questionnaire accurately. As one provider stated, “Some patients need a little guidance to make sure they answer correctly” (HCP1). Executives emphasized that patient comprehension was critical not only for ensuring valid data but also for practical implementation. One executive highlighted that differences in education levels could affect how patients interpret items, noting that “if patients are less educated, they may not understand what goals are” *(Executive2),* requiring additional grouping and support strategies to ensure meaningful responses.

#### 3.4.2 Sub-theme 1.2: functional ability and independence

Most participants reported being able to complete the questionnaire independently, reflecting adequate vision, mobility, and familiarity with written forms. However, physical limitations—particularly those associated with aging—were identified as important barriers. For example, one patient explained, “I can’t see it clearly; my eyesight is not good. I can’t do it myself” (Patient 3), illustrating how visual impairment limited independent completion.

Healthcare providers similarly reported that additional support was sometimes required, particularly for older adults or patients with cognitive impairments. As one provider noted, “For patients with memory problems, we had to read questions aloud and clarify options” (HCP3), highlighting the need for interviewer assistance in certain cases. From an executive perspective, these practical challenges were considered critical for successful implementation, with one executive emphasizing the need to “plan for support staff or alternative formats for those who can’t complete it themselves” (Executive1).

#### 3.4.3 Sub-theme 1.3: perceived value

Patients perceived value of the PREM strongly influenced their engagement and motivation to provide thoughtful responses. Many participants viewed the PREM not merely as a questionnaire but as an opportunity for reflection and learning. For example, one patient explained that “it helps me understand more about diabetes, such as how to use my medication properly” (Patient 1), while another described how it reinforced prior education, noting that “it helps me review what the healthcare staff has taught, like managing blood glucose. It also stimulates my brain to remind myself” (Patient 4).

Several participants emphasized the PREM's role in reinforcing self-care behaviors and treatment adherence. One participant stated that it “reminds me of my medication and following the doctor's advice,” including healthy eating and foot and eye care (Patient 9). Others highlighted its broader educational value, suggesting that it could increase awareness as “diabetes becomes more common in the community” (Patient 8). Some also perceived it as a reminder of key topics that healthcare providers should address during consultations, with one participant noting that “it reviews what the doctor or nurse should do” (Patient 7).

Healthcare providers similarly perceived the PREM as a useful tool to facilitate patient expression and enhance communication about care experiences. As one provider explained, “it's beneficial because it allows patients to discuss their own treatment and how they’re receiving the service” (HCP3), suggesting that it may encourage more open dialogue. Executives also recognized its strategic value in supporting collaborative care and treatment planning, with one executive stating, “I like the idea of planning treatment and providing information about patients’ illnesses” (Executive3), indicating its potential to guide more patient-centred care.

#### 3.4.4 Sub-theme 1.4: trust in the healthcare environment

Patients' willingness to provide honest and potentially critical feedback was closely linked to their trust in healthcare staff and the clinical environment. When asked whether completing the PREM might affect their relationship with healthcare providers, most participants reported no concern. This confidence was often attributed to trust in staff professionalism and reassurance about anonymity. For example, one participant stated, “I'm not worried; it's general feedback and doesn't identify me” (Patient 1), while another emphasized trust in the care team, noting, “No, I trust the professionalism of the healthcare staff” (Patient 6).

Others highlighted the importance of anonymity in enabling open responses. As one patient explained, “I don't worry because the doctor won't know who I am” (Patient 8), suggesting that confidentiality reduced fear of negative consequences. Some participants also framed honest feedback as a personal responsibility, with one stating, “I'm not afraid; I think I should stand for the truth” (Patient 12), reflecting a sense of moral commitment to providing accurate responses.

### 3.5 Theme 2: instrument characteristics

#### 3.5.1 Sub-theme 2.1: desgin and usability

Participants discussed intrinsic characteristics of the PREM, particularly its design, usability, content, and relevance, as important factors influencing feasibility.

Patients described some uncertainty regarding response formats and instructions, highlighting the need for clear guidance. For example, one participant stated, “I'm not sure which channel to choose, but having someone at the hospital to help makes it easier” (Patient 19), while another expressed confusion about response marking, noting, “I don't know clearly where I need to check the mark or answer” (Patient 13). These responses indicate that clear instructions and support are essential for confident completion.

Practical considerations such as questionnaire length and age appropriateness were also raised. Some participants suggested limiting the number of pages, with one noting that “three pages is OK” (Patient 12), while another commented that “the text is long and voluminous. If it is shorter, it is okay” (Patient 9). When asked about preferred mode of administration, most patients indicated a preference for paper-based formats, particularly given the older age profile of clinic attendees.

Healthcare providers similarly emphasized the importance of clarity and accessibility to ensure meaningful responses. They observed that older adults and individuals with limited literacy often struggled with formal language or numerical rating scales. As one provider explained, “Some patients don't fully understand the scoring method, and if the language is too formal, they may not grasp the meaning” (HCP 3). Another suggested using “semi-formal, something that's easy for the elderly to understand” (HCP 1). Executives echoed these concerns, noting that numerical scoring systems could create confusion if not clearly presented.

Format and durability were also discussed. While one provider acknowledged that digital versions “will last longer” than paper (HCP 1), organizational policies currently favor paper use to conserve resources. At the same time, executives stressed that the tool must remain simple and accessible, warning that “if the tool is too complicated, staff have to spend time explaining it instead of focusing on care” (Executive1). Given the large proportion of elderly patients, paper-based administration was viewed as more practical and appropriate.

#### 3.5.2 Sub-theme 2.2: content relevance and applicability

The perceived relevance and applicability of PREM items influenced participants' ability to respond meaningfully. Some patients reported that certain questions did not align with their personal experiences or aspects of their care. For example, one participant stated, “I never talked to my doctor about setting goals, so that question didn't apply to me” (Patient 4), indicating that goal-setting was not routinely discussed in their consultations. Another patient expressed uncertainty about shared target-setting, explaining, “I only know that taking medicine does reduce blood sugar levels, but I am not sure about setting blood sugar targets together with medical personnel” (Patient 5). These responses suggest potential gaps between questionnaire content and patients' lived experiences of care.

Healthcare providers and executives also raised concerns about item clarity and conceptual understanding. One provider anticipated that many patients might select neutral scores due to limited comprehension, noting that “they would score 3, which is average because they don't have any idea how to score it” (HCP 3). Executives further emphasized that certain terms, such as “planning,” may be too abstract for some patients. As one executive explained, “They don't understand what planning is. They just know what their illness is, how to treat it, how to take care of themselves, and how to behave” (Executive 2).

These findings highlight the importance of tailoring PREM items to the local context and ensuring that terminology reflects patients' actual care experiences and level of understanding**.**

### 3.6 Theme 3: contextual and implementation factors

This theme addresses the practical considerations for integrating the PREM into routine clinic workflows, highlighting factors that influence feasibility, timing, and staff involvement.

#### 3.6.1 Sub-theme 3.1: feasibility of routine implementation

The feasibility of routine PREM administration was shaped by patient, staff, and organizational factors. Most patients were able to complete the PREM successfully, particularly when it was integrated into clinic waiting periods. Many preferred completing the questionnaire at the hospital, where assistance was readily available. As one participant noted, “Hospital, because I won't forget to submit the answer” (Patient 1), suggesting that the clinical setting increased accountability. Although most preferred on-site completion, a small number indicated flexibility, with one patient stating, “If not in a hurry, I prefer to do it at home” (Patient 4).

Regarding frequency, most participants considered twice per year (every six months) to be manageable and aligned with routine follow-up schedules. One executive affirmed that “two visits per year would be fine, because patients come in regularly anyway” (Executive 2). However, one patient suggested a more frequent schedule, proposing administration “every 3 months, but ask a new person each round” (Patient 6), indicating variability in preferences.

Healthcare providers highlighted time constraints during busy clinic hours, emphasizing that implementation would only be feasible if integrated into existing waiting periods. As one provider explained, “It's feasible if we integrate it into waiting times, but not during busy clinic hours” (HCP3). Executives similarly stressed the importance of workflow planning, while cautioning that certain waiting periods, such as before pharmacy dispensing, may reduce attentiveness. As one executive observed, “In the end, the patient won't pay attention. They'll just listen for their turn to get their medication” (Executive 1).

Overall, successful integration of the PREM into routine care was viewed as dependent on aligning administration with clinic flow, minimizing time burden, and selecting appropriate timing within the patient visit.

#### 3.6.2 Sub-theme 3.2: policy and organizational support

Executives emphasized that institutional support and policy alignment were critical for the successful adoption of the PREM. They noted that staff engagement often depends on whether management prioritizes the initiative. As one executive stated, “If management values it, staff will make time to administer it properly” (Executive1), underscoring the importance of leadership endorsement in shaping implementation behavior.

Aligning the PREM with existing hospital policies and quality improvement initiatives was also viewed as essential. One executive highlighted the need for formal policy support, stating, “We need policies that support using this tool for patient-centred quality improvement.” (Executive3) Linking PREM administration to dedicated quality improvement teams was suggested as a strategy to improve data accuracy and ensure systematic feedback into service development. For example, one executive recommended that “the research team should collect and analyse the data to ensure objective results” (Executive3), with findings subsequently shared with the NCD service team for improvement.

Practical resource considerations were also raised. Executives acknowledged limitations in IT infrastructure, noting that digital devices such as iPads were unlikely to be available. However, they emphasized that having dedicated personnel to assist with administration would reduce staff burden. As one executive explained, “IT equipment like iPads isn't likely to be available, but having someone to help would be great. It would save staff from overloading their daily work.” (Executive2).

Co-design was further emphasized as a key strategy to enhance relevance and clarity. One executive recommended “co-designing the tool” (Executive1) for future routine use to ensure contextual appropriateness. Additionally, the need for structured support during administration was highlighted, including having designated personnel to read questions aloud, confirm understanding, and assist with interpretation. To minimize bias and enhance objectivity, one executive suggested that those administering the PREM should be external to the hospital, noting, “I'd like to request that outsiders not be hospital staff, as this would create bias. You'll need interns.”(Executive2).

Overall, executives viewed policy endorsement, organizational alignment, dedicated resources, and clear role allocation as central to sustainable PREM implementation.

Taken together, these findings highlight a range of facilitators and barriers to the routine implementation of the PREM across patients, healthcare providers, and hospital executives. A synthesized overview of the key facilitators and barriers identified across patients, healthcare providers, and hospital executives is presented in Table 2.

## 4 Discussion

### 4.1 Principal findings

This study explored the perceived barriers and facilitators to the routine use of PREM in diabetes care among patients, healthcare providers, and hospital executives. The analysis revealed three overarching themes: patient-related factors, instrument characteristics, and contextual and implementation factors. These themes collectively influence the feasibility, acceptability, and perceived value of PREM use in routine practice. These findings highlight that successful implementation depends not only on patient understanding and tool usability but also on institutional readiness and alignment with organizational quality improvement goals.

### 4.2 Patient engagement and equity

Patients' comprehension and health literacy strongly influenced engagement and the accuracy of responses. Complex wording and medical terminology made it difficult for patients to complete the questions meaningfully, particularly for older adults and those with limited literacy. An executive noted, “The term ‘planning’ is too abstract for patients. They don't understand what it means. They only know their illness, how to treat it, how to take care of themselves, and how to behave.” This aligns with prior research underscoring the role of language simplicity and contextual adaptation in ensuring equitable participation in patient-reported instruments (28).

Importantly, the comprehension challenges identified in this study may reflect multiple interacting factors. While differences in health literacy likely contributed to some difficulties, certain PREM items, particularly those referring to abstract concepts such as “planning” or “goal-setting,” may also pose design-related challenges. Moreover, patients' uncertainty regarding these concepts may not solely indicate limited literacy but could suggest that structured care planning and shared decision-making are not consistently emphasized or explicitly communicated during routine consultations. Thus, the observed uncertainty may represent not only a measurement issue but also an opportunity for the clinic to strengthen patient–provider communication and clarify collaborative care processes.

Functional abilities also shaped participation. Patients with visual impairment, memory difficulties, or limited dexterity often require guidance or larger font sizes, consistent with prior studies emphasizing adaptations to promote meaningful engagement in self-reported measures (29). Motivation was further influenced by patients' perceived value of the PREM; participants expressed that providing feedback allowed reflection on health status and reinforced self-management behaviours, consistent with findings from other chronic disease contexts where patient-reported measures supported behaviour change and adherence (18, 30). Trust and perceived confidentiality were additional facilitators. Patients felt more comfortable providing honest feedback when confident in staff professionalism and anonymity, reflecting a psychologically safe care environment that has been shown to enhance response quality (31).

### 4.3 Instrument flexibility and tailoring

Findings related to the intrinsic qualities of the instrument underscore the necessity of flexibility and tailoring. Patient feedback on questions lacking content relevance and applicability (e.g., questions on collaborative goal-setting not applying to all patients) directly aligns with global calls for disease-specific and contextually relevant instruments (31). This suggests that overly standardized items risk collecting inaccurate or incomplete data, supporting the executive view that the PREM should be tailored to diverse patient subgroups.

Findings related to patients' uncertainty or difficulty selecting response options have implications for instrument validity. When items are not fully understood, measurement error may occur, affecting data accuracy. Introducing an additional response option, such as “I do not understand” or “Not applicable,” could help distinguish comprehension difficulties from neutral responses. However, any modification would require further psychometric evaluation to ensure reliability and validity are maintained.

Format preferences were also influential. Many patients, particularly older adults, preferred paper-based questionnaires, describing them as “easier to read” and “less confusing,” while providers valued electronic administration for efficiency and data management. These observations highlight a tension between digital transformation goals and patient capacities (32), underscoring the need to offer multiple formats to ensure equitable participation (33). In clinical settings serving older or less digitally experienced populations, maintaining paper-based options remains crucial for promoting equitable participation and preventing new barriers to access. This observation aligns with previous research emphasizing that digital health innovations must be adapted to users' technological readiness and contextual realities to achieve meaningful engagement (29). Furthermore, providers viewed flexible administration formats and the option for staff support as important facilitators, especially for patients with limited literacy or functional limitations. However, while flexibility and tailoring enhance relevance and inclusivity, a core set of standardized items must be retained to preserve the psychometric robustness of the PREM, including its reliability and validity across settings and populations (34). The PREM used in this study has already undergone testing to establish these psychometric properties (17).

### 4.4 Feasibility and organizational commitment

The feasibility of integrating the PREM into routine clinical workflows appears to depend largely on strategic timing and adequate resource allocation. Both patients and healthcare providers suggested that completion during waiting periods was most practical; however, attention waned toward the end of visits, indicating that administration may be optimal immediately post-consultation. This aligns with prior research advocating for embedding patient-reported measures within natural care processes to maximize engagement and data quality (35).

Institutional support emerged as a critical enabler. Executives emphasized aligning PREMs with hospital policies and quality improvement initiatives, and suggested using external or dedicated staff, such as interns, to facilitate administration. This approach addresses staff workload concerns while improving data objectivity. Executives also highlighted the importance of a structured workflow and suggested that co-designing the PREM with end-users could help ensure clarity, relevance, and smoother integration into routine practice.

All these findings are consistent with prior studies on patient-reported experience measures in chronic care, which identify literacy, instrument usability, and organizational readiness as key determinants of success (29, 36, 37). Importantly, this study provides a context-specific understanding of PREM implementation in Thai diabetes care and highlights practical strategies, such as employing dedicated staff to reduce bias and workload. This operational approach offers empirical support for prior theoretical recommendations aimed at minimizing organizational barriers to PREM use.

Patient comprehension, instrument design, and organizational support were found to interact synergistically. Even a well-designed PREM may fail if patients cannot understand it or if staff are not available to assist, highlighting the importance of a holistic approach that considers individual, instrument, and contextual factors together. Coordinated attention to these factors is essential to optimize feasibility, acceptability, and meaningful data collection.

### 4.5 Practical implications

Implementing PREMs in routine diabetes care should focus on strategies that enhance patient engagement and ensure feasible administration. Questionnaires should be tailored to patients' literacy and cognitive abilities, and multiple formats (paper and digital) should be offered to accommodate diverse preferences. Staff support may be needed to guide patients, particularly older adults or those with functional limitations, while minimizing disruption to clinical workflows. PREM administration should be integrated into natural care processes, such as during waiting periods or immediately post-consultation, to optimize response quality. These strategies collectively support equitable participation and actionable feedback for quality improvement initiatives. At an organizational level, supportive leadership, clear workflow structures, and appropriate allocation of human resources, whether internal or external, are essential to sustain routine PREM use.

Although this study was not designed as a validation study, the qualitative findings complement prior psychometric testing by providing contextual insights into item clarity, response interpretation, and practical feasibility. These findings may inform future refinement of the instrument while preserving its established reliability and validity.

### 4.6 Strengths and limitations

This study has several strengths. By including multiple participant groups, it captured a range of perspectives and provided a comprehensive understanding of factors influencing PREM implementation. The use of all stakeholder interviews allowed exploration of both individual experiences and institutional viewpoints. In addition, including participants' quotations throughout the analysis enhanced transparency and confirmability of the findings. Importantly, the study provides context-specific insights from a Thai primary care setting, addressing the existing knowledge gap on PREM implementation in low- and middle-income countries and informing potential adoption in similar Southeast Asian contexts.

However, the study also has some limitations. Data were collected from a single hospital, which may limit the generalizability of the findings to other healthcare settings. Although transcripts were translated into English for analysis, some nuances may have been lost during translation. Finally, as a qualitative study, the findings are context-specific and exploratory, highlighting the need for further research to assess the prevalence and impact of the identified barriers and facilitators in broader populations.

## 5 Conclusions

This study provides context-specific insights into the factors influencing the routine use of PREMs in Thai diabetes care. Patient comprehension, instrument design and flexibility, and organizational support jointly determine the feasibility, acceptability, and perceived value of PREM implementation. Addressing these factors through tailored questionnaires, supportive administration, and institutional commitment can enhance the systematic collection of patient-reported experiences, promote patient-centred care, and inform broader adoption and implementation strategies in similar settings.

## Data Availability

The raw data supporting the conclusions of this article will be made available by the authors, without undue reservation.
