# Supplementary material for: Barriers and facilitators to using patient-reported experience measures for diabetes care: a qualitative study in Thailand
Source: Front Health Serv. 2026 Apr 10;6:1799682. doi: 10.3389/frhs.2026.1799682 (PMC13106585; doi:10.3389/frhs.2026.1799682)
Supplement: Supplementary file 1 [file Table1.docx]

**แนวทางการสัมภาษณ์เชิงลึกกับผู้ป่วย**

**(10 นาทีเริ่มไปถามคนไข้ พอครบ 20 นาทีควรเสร็จ ถ้าระหว่างนี้ทำไม่ได้จะช่วยอ่านให้และถามให้ตอบ และบันทึกไว้ คนที่มีสิทธิ์จะช่วยคือครอบครัว หรือผู้ช่วยนักวิจัย แจ้งให้คนไข้ทราบว่า แบบสอบถามนี้จะไม่ระบุตัวตน)**

**หมายเหตุ : จับเวลาเริ่มต้น…………..สิ้นสุด…………..วันที่สัมภาษณ์……………………โดย………………..**

- ท่านคิดว่าแบบสอบถามที่ท่านเข้าร่วมก่อนหน้านี้ เข้าใจง่ายหรือยาก? อย่างไร? หากผู้ป่วยตอบว่า **“ง่าย/ยาก**” ให้ถามเพิ่มเติมว่า **เพราะอะไร?**
- ในกรณีที่ตอบว่า **“ยาก”** ท่านคิดว่าคำ (wording)/ ประโยค / คำถาม ไหนในแบบสอบถาม ที่อ่านรู้สึกสับสน ไม่เข้าใจ ไม่ชัดเจน? ตรงส่วนไหน? อย่างไร
- ตอนทำแบบสอบถามเมื่อสักครู่ ท่านสามารถทำได้เองหรือไม่? อย่างไร?
- ท่านคิดว่าเข้าใจแบบสอบถามนี้หรือไม่? อย่างไร? ระดับไหน? (น้อย ปานกลาง มาก) เพราะอะไร?
- *ถ้าหากผู้ป่วยไม่สามารถตอบคำถามได้ ให้ถามแบบชี้นำ* เช่น รู้สึกสับสนหรือไม่? ข้อไหน? ทำได้ทุกส่วนหรือไม่? ถ้าทำไม่ได้ส่วนไหนที่ต้องให้คนมาช่วย? ใช้เวลาทำนานหรือไม่? ท่านมีความพึงพอใจต่อแบบสอบถามนี้ระดับไหน? (ชอบ/ไม่ชอบ - น้อย ปานกลาง มาก) เพราะอะไร? ควรปรับปรุงอย่างไร?
  - ใช้เวลานานแค่ไหน? แบบสอบถามนี้ยาวหรือสั้น? ต้องประมาณไหนถึงจะเหมาะสม?

1. สภาพแวดล้อมของสถานที่นี้ มีผลต่อการตอบแบบสอบถามนี้หรือไม่? อย่างไร? (ควรทำที่ไหน ที่บ้านหรือโรงพยาบาลดีกว่า ช่วงเวลาใด ควรกลับมาทำแบบสอบถามบ่อยไหม ควรมีบุคคลากรทางการแพทย์มาช่วยทำด้วยหรือไม่ หรือไม่ต้องมีดีกว่า) ท่านคิดว่าแบบสอบถามนี้มีประโยชน์หรือไม่? อย่างไร? (ลองตอบดูก่อน**)

- แบบสอบนี้ช่วยในการดูแลโรคเบาหวานของท่านไหม? อย่างไร? *** ขอลงรายละเอียดเยอะๆ***

1. ในอนาคตถ้ามีการใช้แบบสอบถามนี้ ท่านอยากจะใช้ไหม? อย่างไร? ท่านคิดว่าการทำแบบสอบถามนี้จะส่งผลต่อเรื่องความสัมพันธ์ของท่านกับผู้ให้บริการอย่างไร ท่านชอบทำแบบสอบถาม แบบกระดาษหรือแบบออนไลน์? เพราะอะไร?

-------บันทึกพฤติกรรมขณะสัมภาษณ์ด้วย!!!-------

**แนวทางการสัมภาษณ์เชิงลึกกับทีมบุคลากรทางการแพทย์** ในระบบบริการดูแลผู้ป่วยเบาหวานของโรงพยาบาลท่านมีการประเมินความพึงพอใจของผู้ป่วยหรือไม่ ถ้ามีประเมินด้านอะไรบ้าง? หลังจากใช้แบบสอบถามนี้แล้วท่านคิดยังไงกับแบบสอบถาม ด้านไหนที่มีประโยชน์ ยาก/ง่ายหรือไม่?เพราะอะไร?เน้นถามอันใหม่ (เปิดเอกสารไปกับเขา ในมุมมองคนไข้ท่าน*คิด*ว่าแบบสอบถามนี้...)ท่านคิดว่าแตกต่างจากแบบประเมินที่มีอยู่แล้วอย่างไร?

1. ท่านคิดว่ามีปัจจัยภายนอกหรือนโยบายใดที่ส่งผลให้ท่านใช้เครื่องมือนี้? แบบสอบถามนี้สอดคล้องกับความต้องการและความพึงพอใจของผู้ป่วยหรือไม่? อย่างไร?
2. ถ้ามีแบบสอบถามนี้เพิ่มเข้าไปในขั้นตอนการดูแลผู้ป่วยของท่าน ท่านคิดว่าจะมีผลต่อบริการของท่านหรือไม่ อย่างไร?
3. ท่านคิดว่าตอนนี้ทางโรงพยาบาลมีทรัพยากรใด ที่ส่งเสริมหรือสนับสนุนต่อการใช้แบบสอบถามนี้ เช่น เวลา การฝึกอบรมของเจ้าหน้าที่ เทคโนโลยี (อุปกรณ์ การนำแบบสอบถามเข้าไปอยู่ในระบบ รพ)
4. ท่านคิดว่ามีความเป็นไปได้แค่ไหน ที่จะนำแบบสอบถามนี้ไปใช้ในคลินิกของท่าน?
5. ท่านคิดว่าควรใช้กลยุทธหรือแผนใดที่สามารถช่วยนำแบบสอบถามนี้ไปใช้ในคลินิกของท่านได้?

-------บันทึกพฤติกรรมขณะสัมภาษณ์ด้วย!!!-------

**แนวทางการสัมภาษณ์เชิงลึกกับทีมผู้บริหารโรงพยาบาล(Interview guide for Executives group)**

1. ท่านมีมุมมองอย่างไรต่อการนำแบบสอบถามที่ท่านเข้าร่วมก่อนหน้านี้ ใหม่มาใช้ในคลินิก?
2. ท่านประเมินความคุ้มค่าและความเป็นไปได้ในการนำแบบสอบถามที่ท่านเข้าร่วมก่อนหน้านี้ มาใช้อย่างไร?
3. นโยบายการกำกับดูแล หรือแรงกดดันจากภายนอกส่งผลต่อการตัดสินใจนำแบบสอบถามที่ท่านเข้าร่วมก่อนหน้านี้ มาใช้อย่างไร?
4. ณ ตอนนี้ การสนับสนุนจากภายนอก (เช่น ทุน ความร่วมมือ) เพื่อสนับสนุนการใช้แบบสอบถามที่ท่านเข้าร่วมก่อนหน้านี้ มีอะไรบ้าง?
5. ลำดับความสำคัญขององค์กรและการมีส่วนร่วมของผู้นำ ส่งเสริมหรือขัดขวางการนำแบบสอบถามที่ท่านเข้าร่วมก่อนหน้านี้ มาใช้อย่างไร? มีกลยุทธ์อะไรบ้างในการแก้ไขอุปสรรคภายในองค์กร?
6. ท่านประเมินความพร้อมและความเต็มใจของเจ้าหน้าที่ในการใช้แบบสอบถามที่ท่านเข้าร่วมก่อนหน้านี้ อย่างไร?
7. มีการฝึกอบรมหรือการพัฒนาทางวิชาชีพอะไรบ้างที่สนับสนุนเจ้าหน้าที่ในการใช้แบบสอบถามที่ท่านเข้าร่วมก่อนหน้านี้ ?
8. ท่านสามารถอธิบายขั้นตอนการนำแบบสอบถามที่ท่านเข้าร่วมก่อนหน้านี้ มาใช้ในโรงพยาบาลของท่านได้หรือไม่?
9. ท่านคิดว่าต้องมีการปรับปรุงหรือเปลี่ยนแปลงอะไรบ้างเพื่อสนับสนุนการนำแบบสอบถามที่ท่านเข้าร่วมก่อนหน้านี้ มาใช้อย่างมีประสิทธิผล?

-------บันทึกพฤติกรรมขณะสัมภาษณ์ด้วย!!!-------

**Semi-Structured Interview Guide in English**

*The semi-structured interview guide was developed based on existing literature on barriers and facilitators to implementing Patient-Reported Experience Measures (PREMs) in diabetes care. The guide was informed by the* Consolidated Framework for Implementation Research *(CFIR) to ensure comprehensive coverage of relevant implementation domains.*

**A. Patient Interviews**

**1. Intervention Characteristics**

- Was the questionnaire easy or difficult to understand? Why?
- Which words, sentences, or questions were unclear or confusing?
- Was the length appropriate?
- How long did it take to complete?
- What improvements would you suggest?

**2. Characteristics of Individuals**

- Were you able to complete the questionnaire independently?
- How confident did you feel while answering?
- To what extent did you understand it (low, moderate, high)? Why?
- Did any sections require assistance?

**3. Inner Setting (Clinical Context)**

- Did the clinic environment affect your ability to complete the questionnaire?
- Would it be better completed at home or at the hospital?
- Should healthcare staff assist patients?

**4. Outer Setting**

- Do you think this questionnaire reflects your needs as a patient?
- Does it address important aspects of your diabetes care?

**5. Implementation Process and Perceived Impact**

- Do you think this questionnaire is useful? Why?
- Does it help improve your diabetes care? Please explain in detail.
- Would you be willing to complete it in the future?
- Do you think completing it could affect your relationship with healthcare providers?
- How often should it be administered?

**6. Format Preference**

- Do you prefer paper-based or online format? Why?

**B. Healthcare Provider Interviews**

**1. Intervention Characteristics**

- What are your overall impressions of this questionnaire?
- Which aspects are useful?
- Is it easy or difficult to understand?
- How does it compare with existing evaluation tools?

**2. Outer Setting**

- How do external policies or guidelines influence the use of PREMs?
- Do patients' needs or expectations drive the adoption of such tools?

**3. Inner Setting**

- Does your clinic currently use tools to assess patient experience?
- How would integrating this questionnaire affect workflow?
- How does clinic culture influence implementation?
- Are sufficient resources available (time, staff, technology)?

**4. Characteristics of Individuals**

- How confident do staff feel about using this tool?
- Do you foresee any resistance among staff?

**5. Implementation Process**

- How feasible is implementation in your clinic?
- What strategies would support successful implementation?

**C. Hospital Executive Interviews**

**1. Intervention Characteristics**

- What is your overall assessment of this questionnaire?
- Do you consider it valuable?

**2. Outer Setting**

- How do regulatory policies or accreditation standards influence adoption?
- What external funding or partnerships support implementation?

1. **Inner Setting**

- How do organizational priorities influence adoption?
- How ready is the organization to implement this tool?
- Are sufficient resources available?

**4. Characteristics of Individuals**

- How willing are staff to adopt this tool?
- Is training available?

**5. Implementation Process**

- Can you describe the process required to implement this questionnaire?
- What internal barriers need to be addressed?
- What changes are necessary for effective implementation?

-------------------------------------End----------------------------------
